# Transcriptome and Small RNA Sequencing Reveals the Basis of Response to Salinity, Alkalinity and Hypertonia in Quinoa (*Chenopodium quinoa* Willd.)

**DOI:** 10.3390/ijms241411789

**Published:** 2023-07-22

**Authors:** Huanan Han, Yusen Qu, Yingcan Wang, Zaijie Zhang, Yuhu Geng, Yuanyuan Li, Qun Shao, Hui Zhang, Changle Ma

**Affiliations:** 1 College of Life Sciences, Shandong Normal University, Wenhua East Road 88, Jinan 250014, China; hanhuanan13@163.com (H.H.); 17852268051@163.com (Z.Z.); gengyhu@163.com (Y.G.); shaoqun@sdnu.edu.cn (Q.S.);; 2 CAS Center for Excellence in Molecular Plant Sciences, Fenglin Road 300, Shanghai 200032, China

**Keywords:** saline-alkali stress, PEG, abiotic stress response, halophytic crops, DGE, miRNA

## Abstract

Quinoa (*Chenopodium quinoa* Willd.) is a dicotyledonous cereal that is rich in nutrients. This important crop has been shown to have significant tolerance to abiotic stresses such as salinization and drought. Understanding the underlying mechanism of stress response in quinoa would be a significant advantage for breeding crops with stress tolerance. Here, we treated the low-altitude quinoa cultivar CM499 with either NaCl (200 mM), Na_2_CO_3_/NaHCO_3_ (100 mM, pH 9.0) or PEG6000 (10%) to induce salinity, alkalinity and hypertonia, respectively, and analyzed the subsequent expression of genes and small RNAs via high-throughput sequencing. A list of known/novel genes were identified in quinoa, and the ones responding to different stresses were selected. The known/novel quinoa miRNAs were also identified, and the target genes of the stress response ones were predicted. Both the differently expressed genes and the targets of differently expressed miRNAs were found to be enriched for reactive oxygen species homeostasis, hormone signaling, cell wall synthesis, transcription factors and some other factors. Furthermore, we detected changes in reactive oxygen species accumulation, hormone (auxin and ethylene) responses and hemicellulose synthesis in quinoa seedlings treated with stresses, indicating their important roles in the response to saline, alkaline or hyperosmotic stresses in quinoa. Thus, our work provides useful information for understanding the mechanism of abiotic stress responses in quinoa, which would provide clues for improving breeding for quinoa and other crops.

## 1. Introduction

Quinoa (*Chenopodium quinoa* Willd.), a dicotyledonous chenopodiaceae crop, is called the “Mother of Grain”, as its grains contain balanced nutrients, including protein with all essential amino acids, resistant starch, lipids, mineral elements, vitamins, dietary fiber and many functional components such as saponin and polyphenol. Quinoa originates from the Andes, which have poor soil quality and a harsh climate, and thus possesses a higher tolerance to abiotic stresses, such as drought, cold, UV irradiance and salinization, than most crops grown on plains [1,2]. As a facultative halophyte, some quinoa species can tolerate high salinity levels up to about 400 mM NaCl (as found in sea water) [3]. As a drought-tolerant crop, quinoa can grow and produce seed grains in the semi-desert conditions of Chile with less than 200 mm of annual rainfall [4]. Thus, quinoa is not only a great cereal for optimizing the utilization of saline-alkali farmland, but also a good model for improving stress tolerance breeding in other crops.

Recently, salinity, alkalinity and drought have hampered agricultural production all around the world. They sometimes occur separately, but more often in combination [5]. Drought stress harms plants through increasing osmotic pressure. Saline stress additionally causes ion toxicity, arising from high levels of Na^+^ and Cl^−^ [6]. In addition to these toxicities, alkali stress also induces toxic effects due to high pH and CO_3_^2−^ levels [7]. Thus, plants respond to these three types of stresses through some shared and some specific mechanisms. Plants usually respond to drought through closing the stomata, wilting and other behaviors, and increasing cell osmotic pressure through accumulating carbohydrates [8]. When suffering from saline stress, plants also remove or store harmful ions using ion pumps [9]. While in a high-pH soil environment, proton pumps in plant root cells and synthesis of organic acids play important roles [10,11]. In addition, many abiotic stresses disturb the normal function of mitochondria and chloroplasts and produce reactive oxygen species (ROS), which can be purified via the oxidation cleaning system or neutralized via reducing substances [12]. Furthermore, many plant hormones, such as ABA (abscisic acid), IAA (indole-3-acetic acid) and so on, are involved in the regulation of these stress responses [13]. As important post-transcriptional regulators, miRNA and other non-coding RNA have also been found to participate in the regulation of these stress responses [14].

Compared with other plants, quinoa displays a higher tolerance to many abiotic stresses. When water is limited, quinoa grows deeper roots, rapidly closes its stomata and produces ROS scavengers, antioxidants (e.g., ornithine and raffinose) and osmoprotectants (e.g., soluble sugars and proline) [4]. Expression analysis revealed that ABA signaling, heat-shock proteins (HSPs) and the biosynthesis of flavonoids are involved in drought response in quinoa [4,15]. When suffering from saline stress, quinoa maintains K^+^ and excretes Na^+^ through various strategies, including Na^+^ sequestration in leaf vacuoles via Na^+^/H^+^ exchanger 1 (NHX1) [16], Na^+^ elimination in roots via the salt overly sensitive 1 (SOS1) pathway [17] and K^+^ retention via high-active H-ATPase [18]. Similar to drought stress, salt stress in quinoa also leads to stomatal closure, decreased photosynthetic efficiency and ROS accumulation [3], which can be relieved through increasing pore density and up-regulating the activity of ROS scavengers such as superoxide dismutase (SOD), peroxidase (POD), ascorbate peroxidase (APX), catalase (CAT) and antioxidants such as rutin [19,20,21]. In addition, expression analyses have indicated that ABA signaling and betaine metabolism may play important roles in the salt stress response in quinoa [22,23]. On the other hand, although alkalization is widespread and often combined with salinization, there is limited information on the alkaline stress response in quinoa. Moreover, thus far, no comparative analysis of responses to salt, alkali and drought in quinoa have been performed.

Here, using high-throughput sequencing, we aimed to analyze the similarities and differences of the molecular responses to salt, alkali and drought at the transcriptional level and the small-RNA-mediated post-transcriptional level in quinoa. Our work will provide a better understanding of the response to alkali, as well as the specific and common mechanisms of saline, alkaline and hyperosmotic stress responses in quinoa, which can provide guidance for the abiotic stress tolerance breeding of quinoa and other crops.

## 2. Results

### 2.1. Response of Quinoa Strain CM499 to Saline, Alkaline and Hyperosmotic Stresses

**Figure 1:**
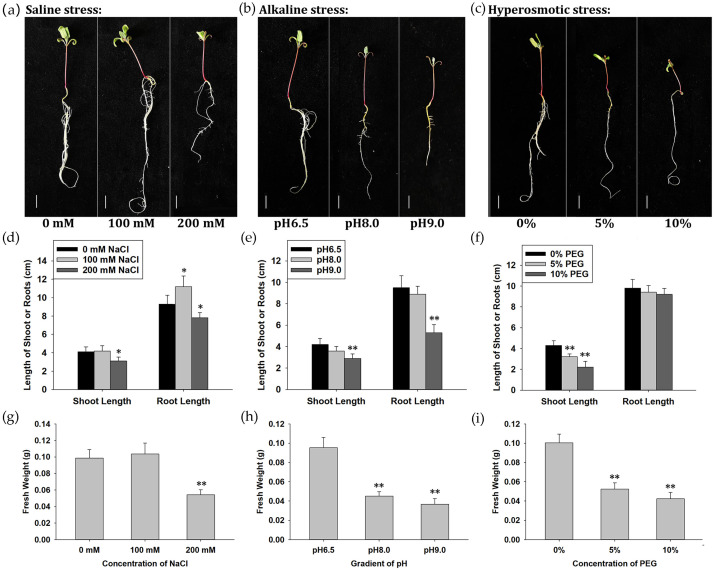
Physiological response of quinoa strain CM499 to saline, alkaline and hyperosmotic stresses. (**a**–**c**) Growth phenotype of CM499 seedlings under saline, alkaline and hyperosmotic stresses, respectively. Scale bars are 1 cm. (**d**–**f**) Statistical analysis of shoot length and root length under saline, alkaline and hyperosmotic stresses, respectively. (**g**–**i**) Statistical analysis of fresh weight per plant under saline, alkaline and hyperosmotic stresses, respectively. The statistical difference between stress treatment groups and control group was analyzed using Student’s T-Test. * means *p* < 0.05 and ** means *p* < 0.01.

As the seedling stage is one of the most sensitive periods to salt and drought in quinoa [4], we transferred 3-day-old seedlings of CM499, a lowland quinoa cultivar suitable for growth in East China (Figure S1), into 1/4 hoagland solution alone (pH 6.5) or with varying concentrations of either NaCl (100 mM and 200 mM), NaHCO_3_/Na_2_CO_3_ (100 mM, pH 8.0 and pH 9.0) or PEG6000 (5% and 10%) to induce saline, alkaline and hyperosmotic stresses, respectively (Figure 1). Five days later, both saline and alkaline stresses led to the abnormal growth of seedlings. In 100 mM NaCl, a slight growth-promoting phenotype was observed, suggesting a certain level of salt tolerance in CM499, while at concentrations of 200 mM NaCl, both shoots and roots became significantly shorter and the fresh weight was decreased (Figure 1a,d,g). Similarly, the growth of the roots was inhibited and the fresh weight was decreased after treatment with alkaline solution, starting at pH 8.0 (Figure 1b,e,h). Effects on shoot and root length were observed at pH 9.0. Seedlings treated with hyperosmotic stress also exhibited phenotypic changes, including a shorter shoot and a lower plant fresh weight, especially under the treatment of 10% PEG. But, noteworthily, the length of roots was not affected so significantly as the ones under saline or alkaline stresses (Figure 1c,f,i). Thus, we inferred that all three stresses might lead to similar physiological responses in seedlings, such as decreased shoot length and fresh weight. But, the growth inhibition of roots might be noticeable under alkaline stress, while the suppression of shoots was obvious under hyperosmotic treatment.

### 2.2. Transcriptome Sequencing of CM499 Seedlings

As the differential phenotype might be the result of early response to treatment of stresses, in this work, we focused on the early transcription response of quinoa seedlings in stress treatment. To compare the molecular responses to saline, alkaline and hyperosmotic stresses in quinoa, we extracted total RNA from 3-day-old CM499 seedlings treated with either 200 mM NaCl, 100 mM NaHCO_3_/Na_2_CO_3_ (pH 9.0), 10% PEG or normal condition (the CK group) for 6 h for high-throughput RNA sequencing (SRA Accession Number: PRJNA994889). There were 42.08–43.58 million clean reads detected in twelve samples (three replicates for the control and three stress treatment groups, respectively), of which 91.38–93.06% were mapped to quinoa genome and 79.63–84.71% to quinoa genes (Table S1). Among these genes, there were 49,138 known genes, of which 11,188 were detected from more than one transcript, and 5485 novel genes (Table S2). The lengths of the identified quinoa genes ranged from 201 bp to 23,672 bp, of which the transcripts were 195 bp to 121,276 bp (but the majority of transcript lengths were less than 10,000 bp). In addition, there were 37,938 genes, all belonging to known genes, with existing functional annotations.

### 2.3. Differential Expression Analysis of Quinoa Genes

**Figure 2:**
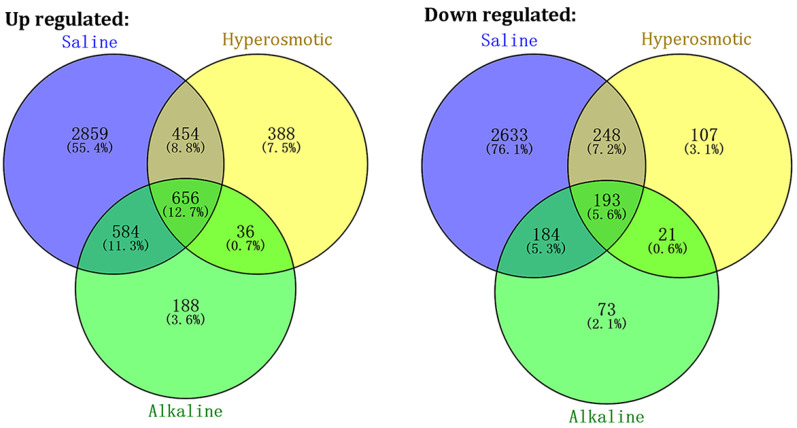
Differential expression analysis of quinoa genes under stress treatment. Genes with obviously altered expression after different stress treatment were counted and displayed as Venn diagram. The left diagram showed the genes that were up-regulated by stresses while the left one showed the down-regulated ones.

Next, we identified the significantly differentially expressed genes (DEGs) whose expression changed significantly after stress treatment compared with the untreated group (the changes were over two-fold and both the *p*-values and the q-values should be under 0.05, Table S3). In total, 8624 genes had significantly altered expression across any of the treatments, among which 5165 genes were up-regulated and 3459 genes were down-regulated (Figure 2). Some of these genes were selected randomly for qRT-PCR and mostly displayed similar trends as the transcriptome, confirming the reliability of the data (Figure S2). Further, we divided these DEGs into seven groups according to the stress they responded to. Over half of the identified DEGs were only found in salt stress, including 2859 genes with increased expression and 2633 genes with decreased expression. On the other hand, 495 DEGs (388 up and 107 down) were only found following PEG treatment and 261 DEGS (188 up and 73 down) were only found following alkaline treatment. There were 1527 DEGs that responded to two stresses, including 702 genes which were significantly altered in both saline and hyperosmotic stress, 768 genes found in both salt and alkaline stress and 57 in both alkaline and hyperosmotic stress. Additionally, 849 genes exhibited significantly changed expression when treated with all three stresses. Thus, the CM499 seedlings seemed to be more sensitive to 200 mM NaCl than 100 mM NaHCO_3_/Na_2_CO_3_ (pH 9.0) or 10% PEG.

### 2.4. Functional Analysis of DEGs in Quinoa Stress Response

**Figure 3:**
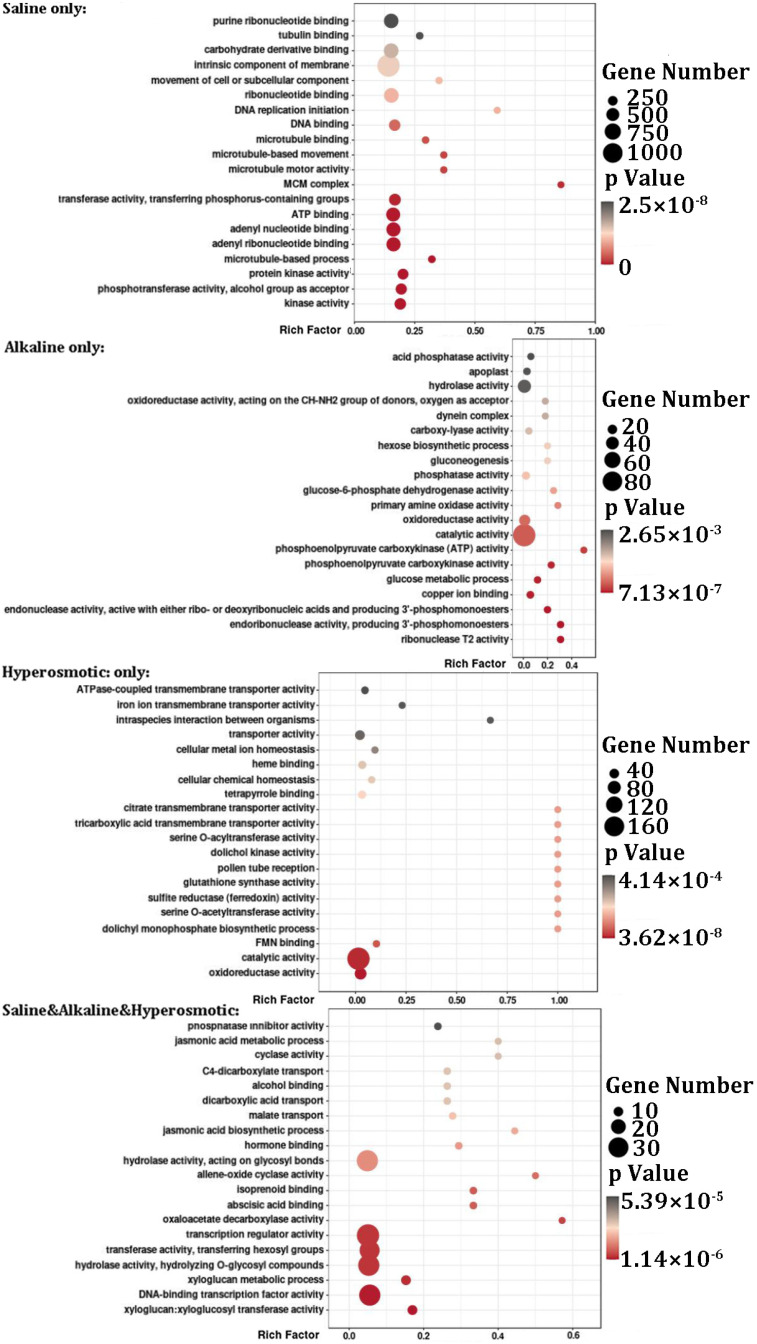
Gene Ontology (GO) enrichment analysis of DEGs in the different stress groups.

Based on their functional annotations, there were 47,751 detected genes which matched to certain GO clusters or KEEG pathways (Table S4). We performed GO enrichment analysis using OmicStudio (https://www.omicstudio.cn/tool, accessed on 4 March 2023) for the seven DEG groups described above (Figure 3 and Figure S3). For DEGs from the “saline only” group, GO terms were enriched for “MCM complex” (complex for the initiation and regulation of DNA replication), “protein kinase”, “phosphotransferase activity” and “ATP binding”. Interestingly, some microtubule-related processes also stood out significantly. For the “alkaline only” group, “endoribonuclease activity”, “phosphoenolpyruvate carboxykinase (ATP) activity” (Gluconeogenesis), ”glucose metabolic process” and “copper ion binding” were enriched. For the “hyperosmotic only” group, oxidoreductase related clusters, “FMN binding”, dolichol related clusters, “serine O-acetyltransferase activity”, “sulfite reductase (ferredoxin) activity”, “glutathione synthase activity”, “pollen tube reception”, “tricarboxylic acid transmembrane transporter activity” and “citrate transmembrane transporter activity” displayed a relative enrichment. For “saline & alkaline” DEGs, “transmembrane transporter activity”, “intrinsic component of membrane”, “integral component of membrane”, “DNA polymerase processivity factor activity”, some “transaminase activity” clusters, “ATP-dependent peptidase activity” and “response to high light intensity” were enriched. For “saline & hyperosmotic” DEGs, “xyloglucosyl transferase activity”, “transcription regulator activity”, “cell wall macromolecule metabolic process”, “apoplast” and “radial pattern formation” were enriched. For DEGs from the “alkaline & hyperosmotic” group, “hydrolase activity, acting on glycosyl bonds”, “actin filament binding”, “xyloglucosyl transferase activity” and “calcium:cation antiporter activity” appeared. For DEGs found in all three stresses (“saline & alkaline & hyperosmotic”), “xyloglucosyl transferase activity”, “oxaloacetate decarboxylase activity”, “abscisic acid binding”, “isoprenoid binding”, “allene-oxide cyclase activity” and “jasmonic acid biosynthetic process” were enriched. Although limited by the *p*-value, there were some clusters not listed in the bubble charts (Table S3); these results still reflected some common and respective characteristics of quinoa in response to the three stresses.

### 2.5. The Acquisition of Small RNA Sequence

**Figure 4:**
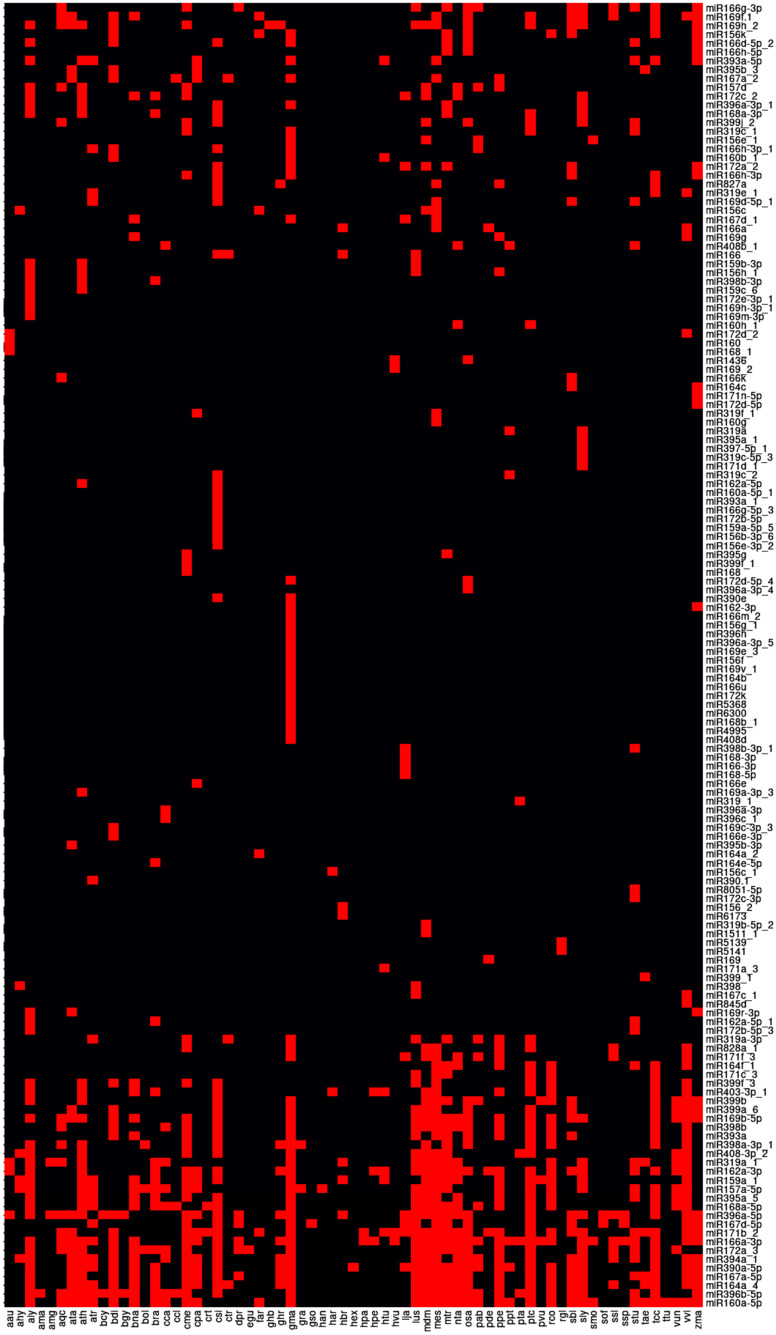
Species conservation analysis of known miRNAs in quinoa. The scientific names of abbreviation are listed in Table S6.

Small RNAs, e.g., miRNAs, are important post-transcriptional regulators in both animals and plants but have not been explored much in quinoa. Using the same samples as used for transcriptome sequencing (three repeats for normal, saline, alkaline and hyperosmotic stresses, respectively), we analyzed small RNAs expressed in quinoa through sequencing the limited length components of the total RNA (18–30 nt). In total, 23.2–24.7 million raw reads were obtained from 12 samples, consisting of 21.9–24.0 million clean reads (SRA Accession Number: PRJNA994977). In the clean reads, the lengths ranged from 18 to 30 nt, with the majority being 24 nt (Table S5; Figure S4). Removing intergenic, intron, exon, unmapped sequences and other small RNA, such as tRNA and rRNA, 0.23–0.55 million reads mapping to miRNA were obtained (Table S5). After aligning the sequences in miRBase, there were 148 miRNAs matched to known miRNA families (Table S6), some of which are conserved across many species, e.g., miR166a-3p, miR160a-5p, miR396b-5p, while others were only identified in limited species, e.g., miR6173, miR166e-3p, miR156g (Figure 4; Table S7). In addition, there were 87 novel miRNAs identified by potential stem-loop structures in the precursors (Table S6).

### 2.6. MiRNA Responses to Different Stresses in Quinoa

**Figure 5:**
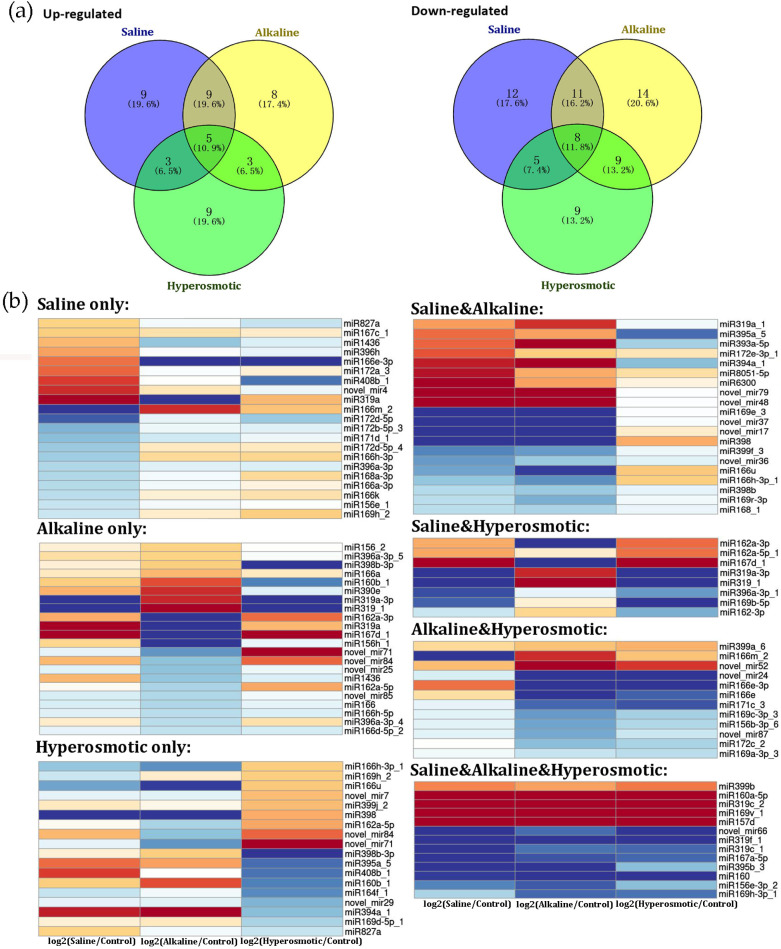
Differential analysis of quinoa miRNAs in saline, alkaline and hyperosmotic stress responses. (**a**) Venn diagram of DEMs. (**b**) Expression heatmap of DEMs belonging to the seven groups divided by the Venn diagram.

To understand the role of miRNAs in quinoa stress responses, differentially expressed miRNAs (DEMs) under saline, alkaline or hyperosmotic stresses were identified as follows: the absolute value of log_2_ (stress treatment/CK) should be over 0.5-fold and both the *p*-values and the q-values should be under 0.05 (Table S8). There were, in total, 114 miRNAs with altered expression under any stress treatment, of which 46 were up-regulated and 68 were down-regulated (Figure 5). Similar to the transcriptomic analysis, we also divided the DEMs into seven groups according to stress types. There were 21 (9 up and 12 down), 22 (8 up and 14 down) and 18 (9 up and 9 down) miRNAs that only responded to saline, alkaline or hyperosmotic stresses, respectively. Additionally, 20 (9 up and 11 down), 8 (3 up and 5 down) and 12 (3 up and 9 down) miRNAs displayed the same expression change trends when treated with saline and alkaline, saline and hyperosmotic or alkaline and hyperosmotic stresses, respectively. In addition, there were 13 miRNAs which responded to all three stresses similarly, including 5 up-regulated and 8 down-regulated ones. Noteworthy, some miRNAs were found to have altered expression under two stresses but in opposing directions (e.g., miR160b_1 was up-regulated under alkaline stress but down-regulated under hyperosmotic treatment), indicating the complex roles of miRNAs in responding to different stresses.

### 2.7. Functional Analysis of DEMs in Quinoa

MiRNAs usually bind to mRNA with a homologous sequence to induce cleavage or inhibit translation of the mRNA. Thus, we predicted the candidate targets of the DEMs to analyze the possible function of these miRNAs in quinoa stress responses. The potential targets of the identified quinoa miRNAs were predicted using TargetFinder and psRobot. In total, 1666 candidate targets (Table S9), belonging to 651 transcripts from 370 genes, were predicted using both two tools (score < 3.5 for TargetFinder, score < 2.5 for psRobot).

**Figure 6:**
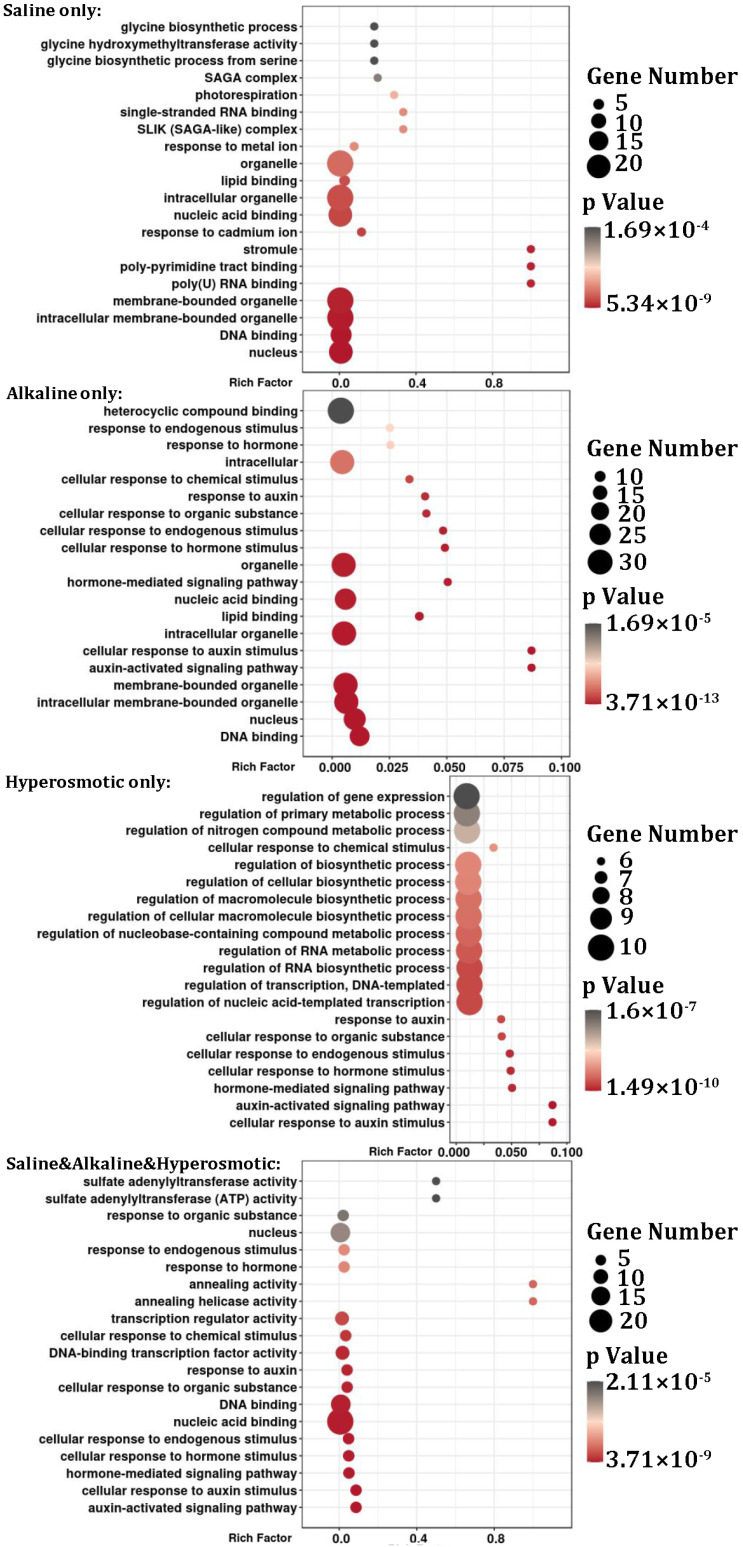
GO enrichment analysis of miRNA targets in quinoa.

According to the stress under which the DEMs were found, the respective targets of the DEMS were also divided into seven groups for GO analysis (Figure 6 and Figure S5; Tables S4, S9 and S10). For “saline only”, “DNA binding”, “poly(U) RNA binding”, “stromule”, “response to cadmium ion”, “lipid binding”, “SLIK (SAGA-like) complex”, “photorespiration” and “glycine biosynthetic process” were enriched. For “alkaline only”, “DNA binding”, “auxin activated signaling pathway” and “lipid binding” were significantly enriched. For “hyperosmotic only”, “auxin activated signaling pathway” and “regulation of transcription, DNA-templated” were enriched. For “saline & alkaline”, “serine family amino acid biosynthetic process”, “sulfate adenylyltransferase activity”, “pyridoxal phosphate binding”, “sucrose alpha-glucosidase activity”, “glycine biosynthetic process” and “DNA-binding transcription factor activity” were enriched. For “saline & hyperosmotic”, “DNA-binding transcription factor activity”, “exonuclease activity”, “sporulation”, “chromosome organization involved in meiotic cell cycle” and “cysteine-type endopeptidase activity” were enriched. For “alkaline & hyperosmotic”, “lipid binding”, “beta-galactosidase activity”, “ferroxidase activity” and “regulation of shoot system development” were significantly enriched. For “saline & alkaline & hyperosmotic”, “auxin activated signaling pathway”, “helicase activity”, “DNA-binding transcription factor activity” and “sulfate adenylyltransferase activity” were significantly enriched.

Some GO clusters appeared in multiple stress groups, but the miRNA–target pairs were rather distinguishable across different groups. For example, auxin-related GO clusters appeared in the “alkaline only”, “hyperosmotic only” and “saline & alkaline & hyperosmotic” groups, which matched to quinoa genes annotated as *ARF* (*auxin response factor*) *17-like* and *ARF18-like* (Figure 6; Table S9). However, these *ARF-like* genes were putatively regulated by miR160b_1 and miR160a-5p (Table S9), the former of which was induced by alkaline treatment but inhibited by hyperosmotic stress, while the latter was up-regulated by all three stresses (Figure 5; Table S8). As another example, the target genes corresponding to the “lipid binding” GO cluster were homeobox-leucine zipper proteins which matched to miR166m_2 in “saline only”, to miR166 and miR166a in “alkaline only” and to miR166e, miR166e-3p and miR166m_2 in “alkaline & hyperosmotic” stresses (Figure 6 and Figure S5; Table S9). For the “sulfate adenylyltransferase activity” GO cluster, the targets were identified as *ATP sulfurylase 1*, which matched to miR395a_5 in “saline & alkaline” and miR395b_3 in “saline & alkaline & hyperosmotic” stresses (Figure 6 and Figure S5; Table S9). For “glycine biosynthetic process”, the targets were *serine hydroxymethyltransferase* (mitochondrial) that matched to miR172d-5p_4 in “saline only” and *serine hydroxymethyltransferase 4* to miR6300 in “saline & alkaline” stresses (Figure 6 and Figure S5; Table S9). These particular examples demonstrated the complexity of the functions of miRNA in saline, alkaline and hyperosmotic stress responses in quinoa.

In addition to the above examples, there were also some other interesting targets matched to the DEMs. Many ethylene-responsive transcription factors, such as *AP2-like*, *RAP2-7-like*, *ERF113-like* and *WIN1-like* were candidate targets of miR172a_3, miR172e-3p_1, miR172c_2 and miR395b_3, which belonged to the “saline only”, “saline & alkaline”, “alkaline & hyperosmotic” and “saline & alkaline & hyperosmotic” groups, respectively (Table S9). Some ubiquitin-related factors, such as “*U-box domain-containing protein 33-like*” and “*probable ubiquitin-conjugating enzyme E2*”, were candidate targets of miR1436, miR399f_3, miR399a_6 and miR399b, which were DEMs of the “saline only”, “alkaline only”, “saline & alkaline”, “alkaline & hyperosmotic” and “saline & alkaline & hyperosmotic” groups. Additionally, many *squamosa promoter-binding-like protein* genes were found as the targets of miR156e_1 and miR156_2, which responded to saline and alkaline stresses, respectively. A *calcium uptake protein* gene was predicted as the target of miR166h-3p_1 and miR166u, which were up-regulated in “hyperosmotic only” but down-regulated in “saline & alkaline”. There were also many epigenetic factors that were found as targets of DEMs, e.g., *histone-lysine N-methyltransferase SUVR4* for miR172-3P_1 in “saline & alkaline” stress, while *SWI/SNF-related matrix-associated actin-dependent regulator* and *histone deacetylase HDT1-like* were found to be targets for miR169h-3p_1, which was differentially expressed in “saline & alkaline & hyperosmotic”. In addition, *kinesin-like proteins*, which are related to microtubules, were targets of miR172d-5p in “saline only” and of miR396a-3p_4 in “alkaline only”.

### 2.8. ROS Accumulation in Quinoa Seedlings under Stress Treatment

**Figure 7:**
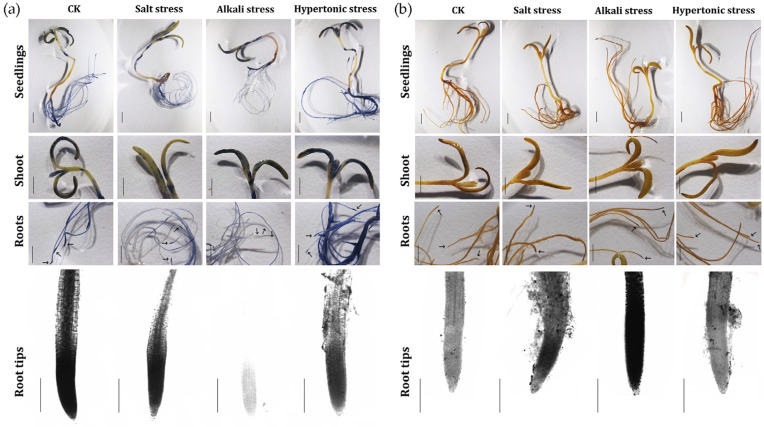
ROS stain of quinoa seedlings under stress treatment. (**a**) NBT stain for CM499 seedlings under stress treatment. (**b**) DAB stain for CM499 seedlings under stress treatment. The upper three rows were seedlings imaged via stereomicroscope while the bottom row was imaged using a digital photographic microscope. The arrows indicate root tips. The bars in the upper three rows are 0.5 cm while the ones in the bottom row are 500 μm.

In plants, ROS are usually produced by the disruption of oxidation–reduction reactions, such as photosynthesis and respiration, induced by abiotic stresses. When under stress treatment, ROS are not only a harmful byproduct that needs to be cleaned, but also an important signal to regulate downstream responses in plants [24]. As our GO analysis displayed, “oxidoreductase activity” was enriched in many DEG groups, including many ROS-related DEGs, such as *respiratory burst oxidase homolog* (*RBOH*), *peroxidase* and *catalase* (Table S3), which indicated a possible role of ROS homeostasis in the quinoa stress response. Thus, we visualized the levels of superoxide and hydrogen peroxide in 5-day-old quinoa seedlings following 1-day stress treatment via staining with NBT (nitrotetrazolium blue chloride) and DAB (diaminobenzidine) stain, respectively. For seedlings under normal cultivation, there was obvious accumulation of superoxide (stood by NBT stain) in the cotyledons, young euphylla, roots and especially the root tips (Figure 7a). When treated with saline stress, the NBT stain was lighter in the cotyledons and roots compared with the control group. The biggest difference was seen in seedlings treated with alkaline stress, in which the root tips were nearly without any stain signal. The seedlings under hyperosmotic stress exhibited a similar NBT stain as the control group, although their root tips showed a slightly lighter coloration. According to the results of DAB staining (Figure 7b), hydrogen peroxide was slightly accumulated in the cotyledons and roots of quinoa seedlings under normal condition and following hyperosmotic stress. However, after being treated with saline stress, the level of hydrogen peroxide increased in the root tips. And even a more greatly increased hydrogen peroxide level was observed in seedlings treated with alkaline stress, which exhibited an almost opposite trend to superoxide. Thus, we concluded that the ROS metabolism was involved in the responses of quinoa to salt and especially alkali. Notably, superoxide and hydrogen peroxide appeared to play different roles in this process, as there was a down-regulation of superoxide but an up-regulation of hydrogen peroxide.

### 2.9. Auxin and Ethylene Were Involved in Quinoa Stress Responses

**Figure 8:**
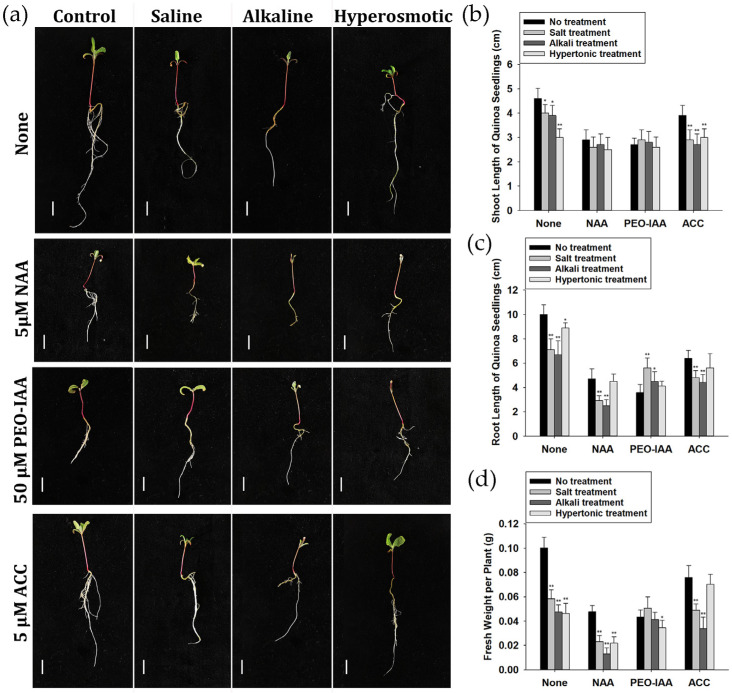
Hormone treatment influenced abiotic stress responses in quinoa. (**a**) Growth phenotype of seedlings following treatment with stresses with and without the addition of hormones. Scale bars are 1 cm. (**b**) Statistical analysis of shoot length. (**c**) Statistical analysis of root length. (**d**) Statistical analysis of fresh weight. The statistical difference between stress treatment groups and normal culture groups was analyzed using Student’s T-Test. * means *p* < 0.05 and ** means *p* < 0.01.

As described in Tables S3 and S9, many ethylene-responsive transcription factors as well as auxin-related genes (e.g., *ARFs*) or their miRNAs displayed altered expression when treated with saline, alkaline or hyperosmotic stresses in quinoa seedlings. To further analyze the relationship between these hormones and stress responses, a series of co-treatment assays were designed (Figure 8). When disturbing endogenous auxin signaling with NAA (naphthylacetic acid) or PEO-IAA (2-(1H-Indol-3-yl)-4-oxo-4-phenyl-butyric acid, an IAA antagonist), the seedlings in the normal culture solution grew more slowly than those with no additives, with shorter shoot length, root length and lower fresh weight. With the addition of NAA, seedlings treated with any of the stresses displayed a significant decrease in shoot length or fresh weight compared with the NAA-treated no-stress group, exhibiting similar alteration trends as those with no additives. After treatment of PEO-IAA, unchanged or increased root length and fresh weight were observed compared to the no-stress, PEO-IAA-treated controls, especially for seedlings treated with saline stress, which was different from those with no additives. In addition, when disturbing endogenous ethylene signaling with ACC (1-aminocyclopropane-1-carboxylic acid, an ethylene precursor), the decrease in root length and fresh weight under hyperosmotic stress was partly alleviated. Thus, our results indicated potential roles of auxin and ethylene signaling pathways in the response to saline and alkaline stresses in quinoa.

### 2.10. Metabolism of Hemicellulose in Quinoa Stress Responses

**Figure 9:**
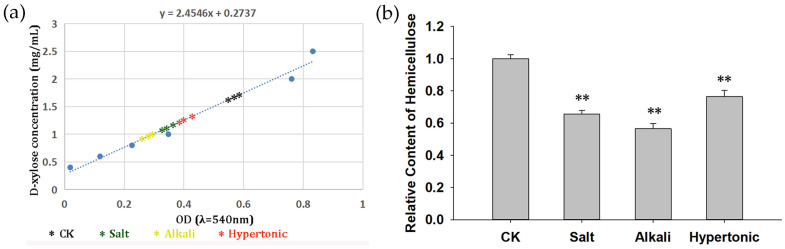
Hemicellulose contents in quinoa seedlings were influenced by stresses. (**a**) Measured values marked in the standard line. (**b**) Relative contents of hemicellulose in quinoa seedlings standardized by the control group (CK). The statistical difference between stress treatment groups and CK groups was analyzed using Student’s T-Test. ** in B means *p* < 0.01.

According to the GO analysis of the DEGs, cell wall synthesis, which is associated with xyloglucosyl transferase activity (xyloglucan endotransglucosylase/hydrolase protein, XTH), was enriched when quinoa seedlings were treated with saline, alkaline and hyperosmotic stresses (Figure 3; Table S3). As the substrate for XTH, xyloglucan is the main type of hemicellulose, of which the content and fiber structure are closely related to cell growth. To clarify the effect of stresses on cell wall synthesis in quinoa, the levels of hemicellulose in seedlings with or without stresses were quantified. The absolute concentration of reducing sugars derived from hemicellulose in quinoa seedlings can be calculated from a standard curve created using D-xylose, and the changed hemicellulose levels between seedlings under normal and stress conditions can be analyzed (Figure 9). Compared with the untreated CK group, the contents of hemicellulose were decreased significantly by all three stresses, with greater effects seen with the saline and alkaline stresses. Therefore, the metabolism of hemicellulose might play a role in the quinoa stress response; however, whether it influences stress tolerance is still unclear.

## 3. Discussion

### 3.1. Plant Hormones Played Important Roles in Quinoa Stress Responses

Plant hormones, such as abscisic acid (ABA), ethylene, auxin and jasmonic acid (JA), regulate not only growth and development, but also stress responses [25,26,27,28,29,30,31,32]. In our transcriptomic data, the GO cluster “abscisic acid binding”, including genes noted as “glutamate receptor” (Figure 3; Table S3), was enriched in the “saline & alkaline & hyperosmotic” group, indicating the involvement of ABA signaling [33]. In the “saline & alkaline & hyperosmotic” DEG group, four allene oxide cyclase (chloroplastic-like) transcripts, belonging to the enriched GO cluster “jasmonic acid biosynthetic process”, were up-regulated by all three stresses (Figure 3; Table S3). MiR6300, induced by both saline and alkaline stresses, was predicted to target *XM_021904655.1*(*LOC110725177*), which was described as *jasmonic acid-amido synthetase JAR1-like* (Figure 5; Table S9). Many auxin-related DEGs, including *auxin-induced proteins*, *auxin-binding proteins*, *auxin-responsive proteins*, *auxin efflux carrier components*, *ARFs*, *auxin transporter-like proteins* and *auxin-repressed proteins*, were distributed across the seven DEG groups, but especially found in the “saline only”, “saline & hyperosmotic” and “saline & alkaline & hyperosmotic” groups (Table S3). In the GO analysis of miRNA targets, the “auxin-activated signaling pathway”, matched to *ARF17* and *ARF18* (paired with miR160b_1 and miR160a-5p, respectively), was enriched in “alkaline only”, “hyperosmotic only” and “saline & alkaline & hyperosmotic” groups (Figure 5a and Figure 6; Table S9). The *auxin signaling F-box 2-like* transcripts, paired with miR393a-5p, were found in the “saline & alkaline” group (Table S9). There were also many ethylene-related DEGs that appeared in all the groups (especially the “saline only”, “saline & hyperosmotic” and “saline & alkaline & hyperosmotic” groups, but absent in the “alkaline & hyperosmotic” group; Table S3), including the *ethylene response factor* (*ERF*), *ethylene insensitive* (*EIN*) and the *ethylene receptor* (*ETR*), which have been associated with responses to salt and drought stresses [34,35,36,37,38,39]. Many *ERFs* (*ethylene response factor*), paired with DEMs such as miR172a_3, miR172e-3p_1, miR172c_2 and miR395b_3, were also found in connection to saline, alkaline and hyperosmotic stresses, respectively (Table S9). In addition, our hormone application assay indicated the possible roles of auxin and ethylene in regulating the stress response of quinoa seedlings (Figure 8). Therefore, the abiotic stress response of quinoa might be closely associated with phytohormones, especially for auxin and ethylene.

### 3.2. Transcription Factors Were Important Regulators of Abiotic Stress Response in Quinoa

Abiotic stress response is a process that integrates different co-factors and signaling pathways to direct a specific action and is usually regulated by transcription factors (TFs) [40,41,42,43]. Our results displayed the enrichment of many GO clusters, such as “DNA binding”, “regulation of transcription” and “DNA-binding transcription factor activity”, in many DEG and DEM groups in various stress treatments (Figure 3; Figure 6; Figure S3; Figure S5). Consistently, there were many TFs, such as *NAC*, *WRKY*, *MYB*, *ZIP*, listed as DEGs or targets of DEMs (Tables S3 and S9). Except for the “hyperosmotic only” and “alkaline & hyperosmotic” groups, we found different quinoa *NAC* genes in the other five DEG groups, most of which were up-regulated by stress treatment, but three (*NAC43-like*, *NAC94-like* and *CUP-SHAPED COTYLEDON 3-like*) were down-regulated in the “saline only” and “saline & alkaline” groups (Table S3). Among these stress-induced quinoa *NAC* genes, *NAC2, NAC17, NAC29, NAC83* and *NAC92* have been found as regulators in response to salt and drought stresses in rice, tobacco, wheat, groundnut and *Arabidopsis* [44,45,46,47,48,49,50]. In addition, miR164f_1, induced by hyperosmotic treatment, was predicted to target *NAC21/22-like* and *CUP-SHAPED COTYLEDON 2-like* genes, which are related to biotic defense and shoot development, respectively [51,52]. Similar to *NAC*, many *WRKY* genes appeared in several DEG groups (Table S3), most of which were induced by different stresses except the following four: *WRKY12-like*, *WRKY13-like* and *WRKY72-like* for “saline only” and *WRKY4-like* for “alkaline & hyperosmotic”. Among these stress-induced *WRKYs*, *WRKY3*, *WRKY20*, *WRKY30*, *WRKY58* and *WRKY75* are associated with stress responses in grape, soybean, *Arabidopsis*, rice and *Solanaceae* plants [53,54,55,56,57]. In addition, there were 37 (24 up-regulated and 13 down-regulated), 1 (up), 6 (5 up and 1 down), 5 (2 up and 3 down) and 5 (all up) *MYB-like* genes in the DEG groups “saline only”, “hyperosmotic only”, “saline & hyperosmotic”, “saline & alkaline” and “saline & alkaline & hyperosmotic” (Table S3), among which *MYB4*, *MYB3R*, *MYB44* and *MYB74* are associated with responses to drought, salt or ABA treatments in *Arabidopsis* and wheat [58,59,60,61]. Several *GAMYB* transcripts were paired with miR319a, miR319a-3p, miR319_1, miR319c_1 and miR319f_1 in the DEM groups “saline only”, “alkaline only”, “saline & hyperosmotic” and “saline &alkaline & hyperosmotic”, respectively (Table S9), which is consistent with the results in rice [62]. Thus, the abiotic stress response of quinoa might be closely associated with TFs.

### 3.3. ROS Metabolism in Abiotic Stress Response of Quinoa

ROS is not only a harmful by-product during stress treatment but also an important molecular messenger in regulating stress response [12]. In our data, the GO cluster “oxidoreductase activity”, which was associated with ROS homeostasis, appeared in all groups and was enriched in the “alkaline only”, “hyperosmotic only” and “alkaline & hyperosmotic” groups (Figure 3 and Figure S3). As the function description exhibited, many *RBOH* members were induced in the “saline only”, “hyperosmotic only” or “saline & alkaline & hyperosmotic” groups (Table S3), suggesting higher production of ROS [63]. Furthermore, many ROS scavengers, e.g., peroxidase and catalase, were also found in the DEG groups “saline only”, “hyperosmotic only”, “saline & alkaline” and “saline & alkaline &hyperosmotic” (Table S3). Thus, we inferred that ROS homeostasis, modulated by oxidoreductases such as RBOHs, peroxidase and catalase, played an important role in quinoa stress responses. In agreement with this hypothesis, NBT and DAB staining displayed changes in ROS accumulation in quinoa seedlings, especially those treated with alkaline and saline stresses (Figure 7). Interestingly, unlike some other crops [64], ROS levels did not increase in the root tips of stress-treated quinoa and even decreased under alkaline treatment, which might indicate the stronger activity of antioxidant enzymes (e.g., peroxidase) than ROS producers (e.g., RBOH). Further, lower superoxide levels appeared with a stronger H_2_O_2_ accumulation, perhaps indicating a relatively lower activity of catalase than peroxidase. Thus, quinoa might have a special ROS homeostasis in abiotic stress responses.

### 3.4. Cytoskeleton Dynamics Might Be Involved in Stress Responses in Quinoa

The cytoskeleton is a dynamic component of plant cells mainly composed of microtubules (MTs) and actin filaments (AFs), the former of which regulate the synthesis of cellulose to microfibrils for cell wall establishment and can effect stomatal movement in response to ABA signaling, while the latter play roles in delivering materials, expanding the cell wall and selectively enabling and/or inhibiting endocytosis and can regulate cell morphogenesis [65,66,67,68]. In our data, some GO clusters such as “microtubule motor activity”, “microtubule-based movement”, “microtubule-based process” and “microtubule binding” were enriched only under saline treatment (Figure 3). These clusters matched to 70 DEGs, mainly including *kinesin-like proteins*, *tubulins* and *microtubule-associated proteins*, surprisingly, all of which were down-regulated by saline stress (Table S3), perhaps indicating a functional inhibition of MTs by saline stress in quinoa. Kinesin is a motor protein which binds to MTs to transport intracellular materials and has been found as a regulator of drought stress response in *Arabidopsis* [69], while in our data the *kinesin-like protein KIN-6* was predicted as the target of miR172d-5p, a DEM in the “saline only” group (Table S3). Some AF-related clusters, e.g., “actin filament binding”, “actin filament-based process” and “actin cytoskeleton organization”, also appeared in the DEG groups “saline & alkaline”, “alkaline & hyperosmotic”, “saline & hyperosmotic”, “saline & alkaline & hyperosmotic” and mainly in the “saline only” group (Figure 3 and Figure S3). AF-related DEGs included *protein-tyrosine-phosphatase MKP1-like*, *actin-depolymerizing factor*, *fimbrin-like*, *SDA1 homolog*, *myosin-like*, *F-actin-capping protein* and *glucan 1,3-beta-glucosidase A* (Table S3), some of which have been found as regulators in stress response [70]. Thus, in quinoa, cytoskeleton-related functions were possibly modified through stress treatment, especially under saline stress.

### 3.5. Cell Wall Organization Was Associated with Stress Response in Quinoa

The plant cell wall is a protective barrier composed of cellulose, hemicellulose, pectin, structural proteins and lignin, which can determine cell size and shape through controlling cell expansion, and is associated with the response to drought and salt in plants [71]. In our work, we found changed levels of hemicellulose in quinoa seedlings under stress treatment (Figure 9), which might be associated with the modulation of the cell wall. There were many GO clusters, such as “xyloglucosyl transferase activity”, “xyloglucan metabolic process” and “cell wall biogenesis”, enriched in the DEG groups “saline & alkaline”, “saline & hyperosmotic”, “alkaline & hyperosmotic” and “saline & alkaline & hyperosmotic” (Figure 3 and Figure S3). These clusters mainly contained DEGs characterized as xyloglucan endotransglucosylase/hydrolases (XTHs), nearly all of which were suppressed by the stresses (Table S3). XTHs can split and reconnect xyloglucan to cellulose microfibrils, mediating the remodification of the cellulose/hemicellulose framework in the cell wall [72]. As a family with dozens of members, XTHs have complex effects on plant stress response. Over-expression of the hot pepper *CaXTH3* enhances tolerance to salt and drought in *Arabidopsis* [73], while AtXTH30 negatively affects the salt tolerance of *Arabidopsis* [74]. In summary, in quinoa, the expression of many *XTHs* as well as the synthesis of hemicellulose were influenced by saline, alkaline and hyperosmotic stresses, which might ultimately influence the cell wall assembly.

## 4. Materials and Methods

### 4.1. Plant Materials and Growing Conditions

Seeds of CM499 were germinated on wet filter paper at 22 °C for two days, then transferred into 1/4 hoagland for hydroponics. Three-day-old seedlings were transferred into 1/4 hoagland with no treatment or stress treatment for phenotypic observation, RNA extraction, or hemicellulose determination. The stress treatments included NaCl (100 and 200 mM), NaHCO_3_/Na_2_CO_3_ (100 mM, pH 8.0 and pH 9.0) or PEG6000 (5% and 10%). For hormone treatment assays, the seedlings were transferred into 1/4 hoagland with no treatment, NaCl (200 mM), NaHCO_3_/Na_2_CO_3_ (100 mM, pH 9.0) or PEG6000 (10% W/V), and each exposure condition was treated with either no additive, 5 μM NAA (CN7541, Coolaber, Beijing, China), 50 μM PEO-IAA (P878117, Macklin, Shanghai, China) or 5 μM ACC (A3903, Sigma-Aldrich, St. Louis, MO, USA). The culture conditions were 22 °C and 16 h-day/8 h-night in an illuminating incubator.

### 4.2. Growth Phenotype Observation

For detection of quinoa’s physiological response to the various stresses, five plants per treatment were cultured in stress solutions for 5 days. Subsequently, the shoot length, root length and fresh weight of each plant was measured. For hormone treatment assays, after treatment with both the hormone and stress for 5 days, the shoot length, root length and fresh weight of five plants for every group were measured.

### 4.3. Transcriptome and Small RNA Sequencing

Seedlings (20 plants per group) treated for 6 h under the various stresses (C1, C2, C3 for normal condition as the control groups; S1, S2, S3 for NaCl treatment as the saline groups; A1, A2, A3 for Na_2_CO_3_/NaHCO_3_ treatment as the alkaline groups; P1, P2, P3 for PEG treatment as the hyperosmotic groups) were collected for total RNA extraction (TRIzol method, BGI, Shenzhen, China) and construction of the respective libraries for transcriptome (6GB per sample) or small RNA sequencing (20 MB per sample, PE150, Illumina HiSeq 2000 device, BGI, Shenzhen, China). Raw reads were initially edited with the Fastx-toolkit pipeline to remove adapter sequences, low quality reads, repetitive reads and, for small RNA, reads longer than 30 nucleotides or shorter than 18 nucleotides. The resulting reads were aligned against the quinoa expressed sequence tag database (https://ftp.ncbi.nlm.nih.gov/genomes/all/GCF/001/683/475/GCF_001683475.1_ASM168347v1, accessed on 5 March 2021), the genomic sequence database and the Rfam database for removal of rRNA, tRNA, snRNA and snoRNA sequences and identification of transcripts. For small RNA sequencing, the reads were compared using BLAST analysis against the miRbase (www.mirbase.org, accessed on 6 March 2021) to identify known miRNAs. Potential novel miRNAs were identified from the remaining reads via miRA (https://github.com/mhuttner/miRA, accessed on 7 March 2021), which could screen miRNA precursors through exploring the characteristic hairpin structure [75]. Target prediction of the identified quinoa miRNAs was made via both TargetFinder and psRobot [76,77], with the default parameters (psRobot: -gl 17 -p 8 -gn 1, TargetFinder: -c 4). The sequence data of transcriptome and small RNA library was deposited in NCBI Sequence Read Archive (SRA, www.ncbi.nlm.nih.gov/sra, accessed on 15 July 2023), with accession numbers PRJNA994889 and PRJNA994977, respectively.

### 4.4. qRT-PCR Analysis

The total RNA from seedlings treated under various stresses (same as the samples for sequencing) for 6 h was extracted (FastPure Plant Total RNA Isolation Kit, RC411-C1, Vazyme, Nanjing, China) and used for reverse transcription (EasyScript One-Step gDNA Removal and cDNA Synthesis SuperMix, AE311-02, Transgen, Beijing, China). An amount of 0.5 g fresh seedlings of each sample was ground in liquid nitrogen, and we extracted the total RNA according to the kit instruction. For each sample, 1 μg total RNA was reverse transcripted to cDNAs following the description of the kit instruction. qRT-PCR was performed via the SuperReal PreMix Plus (SYBR Green) kit (FP205, TIANGEN, Beijing, China) and a real-time PCR detection system (480II, Roche, Switzerland). Quinoa *CqTUB-6* (*LOC110711758*) was used as a reference gene. Abundances were normalized using the 2^−ΔΔCt^ method. The primers used were listed in Table S11, and synthesized by Beijing Tsingke Biotech Co., Ltd. (Beijing, China).

### 4.5. ROS Stain

Five-day-old seedlings (5 plants per group) treated under various stresses (same as the samples for sequencing) for 24 h were submersed in 0.5 mg/mL NBT (CN7731, Coolaber, Beijing, China) within 10 mM Na_2_HPO_4_/NaH_2_PO_4_ (pH 7.6) or 1 mg/mL DAB (CD4181, Coolaber, Beijing, China) solutions (pH 5.8) for 3–8 h in darkness at 28 °C. Then, the seedlings were soaked in 80% ethanol, boiled for 10 min and then imaged via camera and stereomicroscope.

### 4.6. Hemicellulose Determination

Three-day-old seedlings (20 plants per group) treated under the various stresses (same as the samples for sequencing) for 3 days were collected and dried to constant weight in a bake oven at 65 °C. The samples were then ground and filtered using a sifter of 30–50 mesh. The powder was then processed using a Hemicellulose Content Detection kit (BC4445, Solarbio, Beijing, China), following manufacturer guidelines. The hemicellulose was hydrolyzed to reducing sugars, and then reacted with 3,5-dinitrosalicylic acid (DNS) to form a colored product, which could be detected at 540 nm via a spectrophotometer (UV-1800, SHIMADZU, Kyoto, Japan). The relative contents were calculated via a standard curve of various concentrations of xylose and standardized according to the control group.

## 5. Conclusions

Quinoa is a high-nutrient crop with broad stress tolerance. Our work analyzed its response to salt, alkali and hypertonic stresses at the transcriptome and small RNA levels, and we identified a series of stress-responsive genes and miRNAs. The function analysis of DEGs and DEMs revealed factors or pathways related to these stress responses, including plant hormones, TFs, ROS homeostasis, cytoskeleton dynamics and cell wall assembly. We further found that the signaling of ethylene and auxin, the accumulation of superoxide and hydrogen peroxide and the metabolism of hemicellulose were closely associated with the abiotic stress response of quinoa seedlings. In summary, quinoa perhaps shares many common mechanisms in response to saline, alkaline and hyperosmotic stresses, such as xyloglucan metabolism, and possesses some unique pathways such as ROS homeostasis under alkaline stress or cytoskeleton dynamics under saline stress. Our results would afford useful clues for understanding the mechanism of stress response in quinoa.

## Data Availability

The data supporting the conclusions of this manuscript have been displayed as figures and supplemental material, and will be made available by the authors, without undue reservation, to any qualified researcher.

## Supplementary Materials

The supporting information can be downloaded at: https://www.mdpi.com/article/10.3390/ijms241411789/s1.
