# Supplementary material for: Transcriptome and Small RNA Sequencing Reveals the Basis of Response to Salinity, Alkalinity and Hypertonia in Quinoa (Chenopodium quinoa Willd.)
Source: Int J Mol Sci. 2023 Jul 22;24(14):11789. doi: 10.3390/ijms241411789 (PMC10380837; doi:10.3390/ijms241411789)
Supplement: Supplementary file 1 [file ijms-24-11789-s001.zip › Supplement information Han et al. revised.pdf]

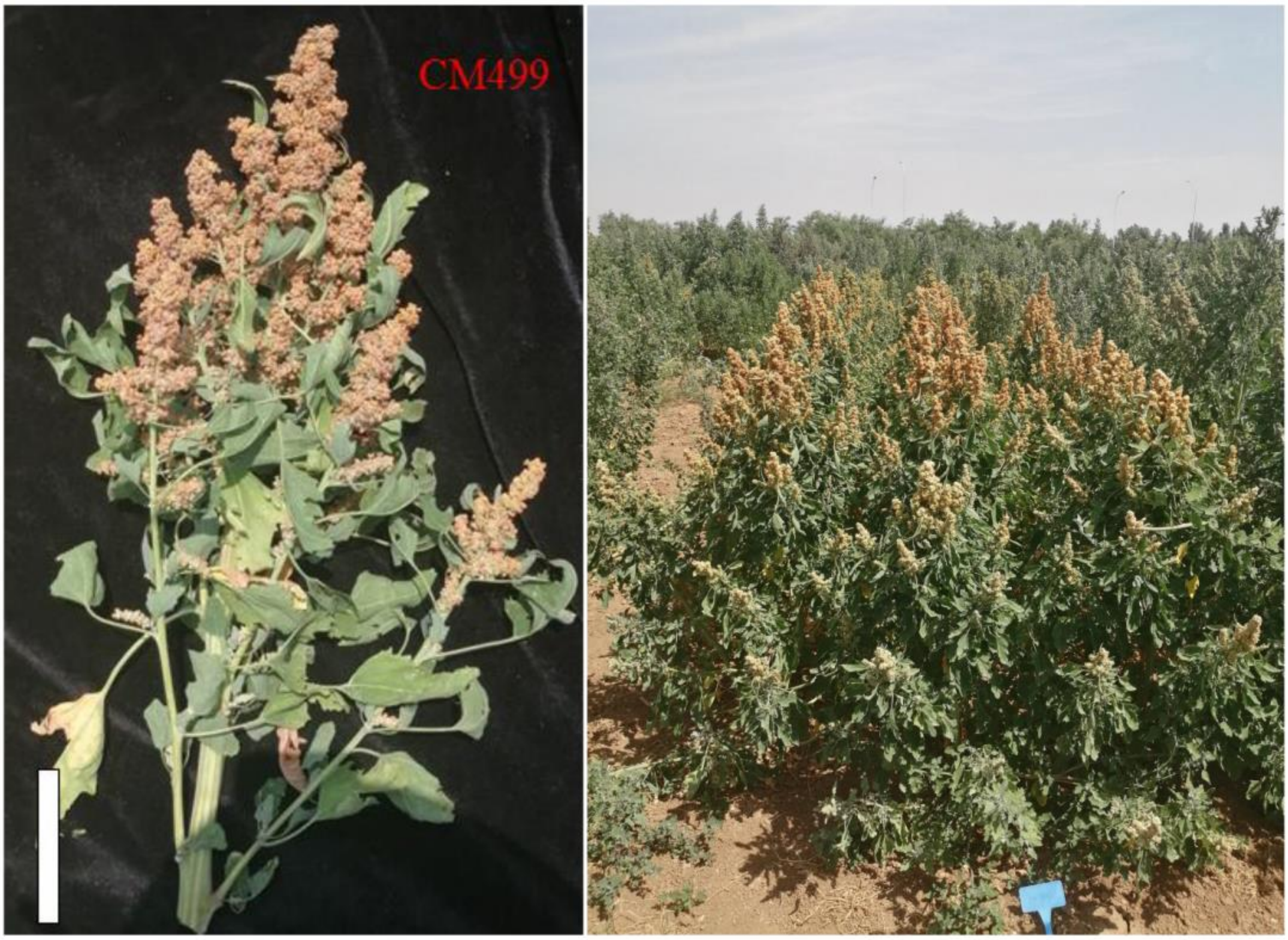

Figure S1. Field phenotype of CM499. It was planted in Zhoucun District, Shandong Province, East China. Bar stood for 10 cm.

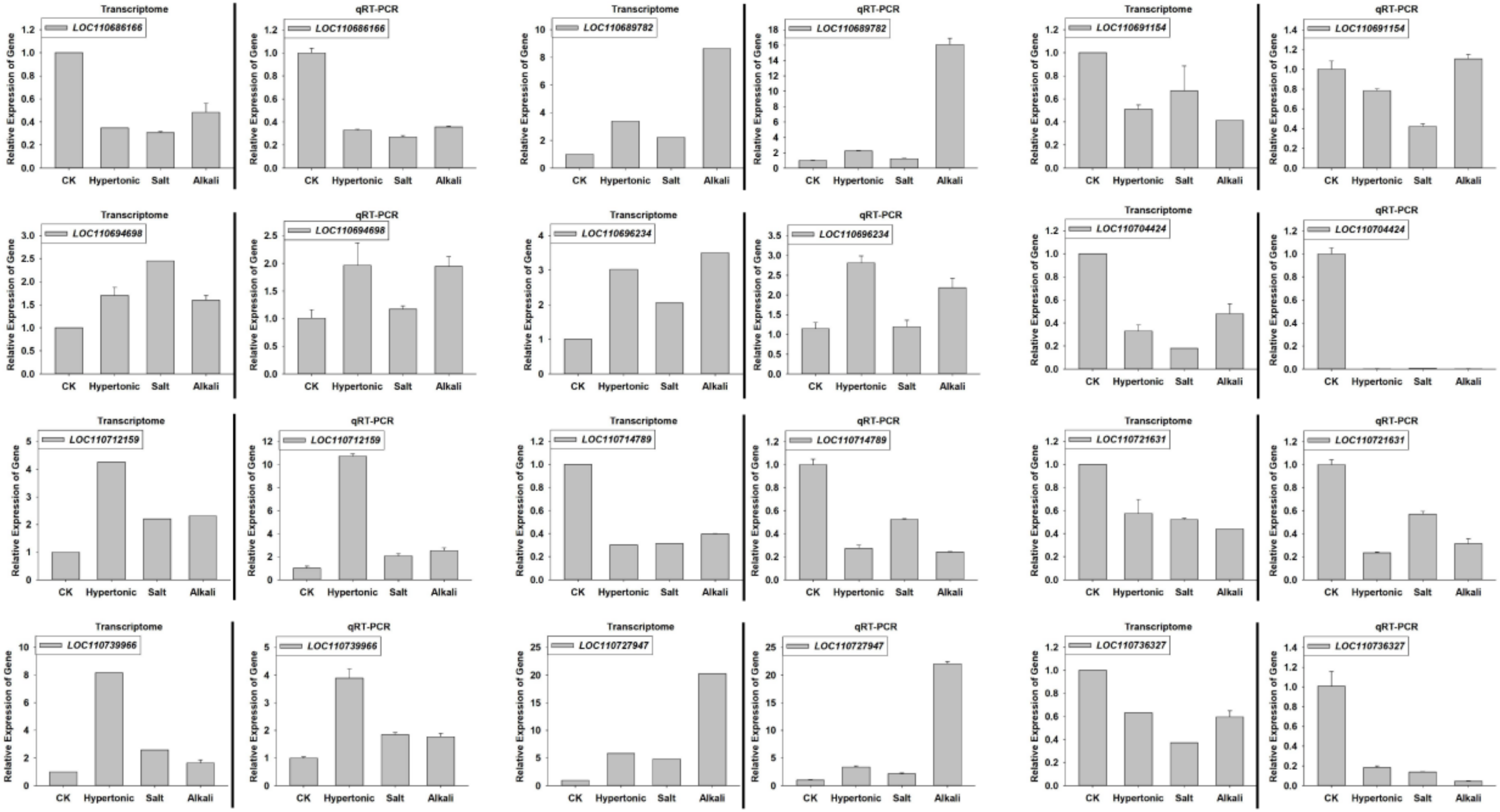

Figure S2. Expression analysis of quinoa genes via qRT-PCR.

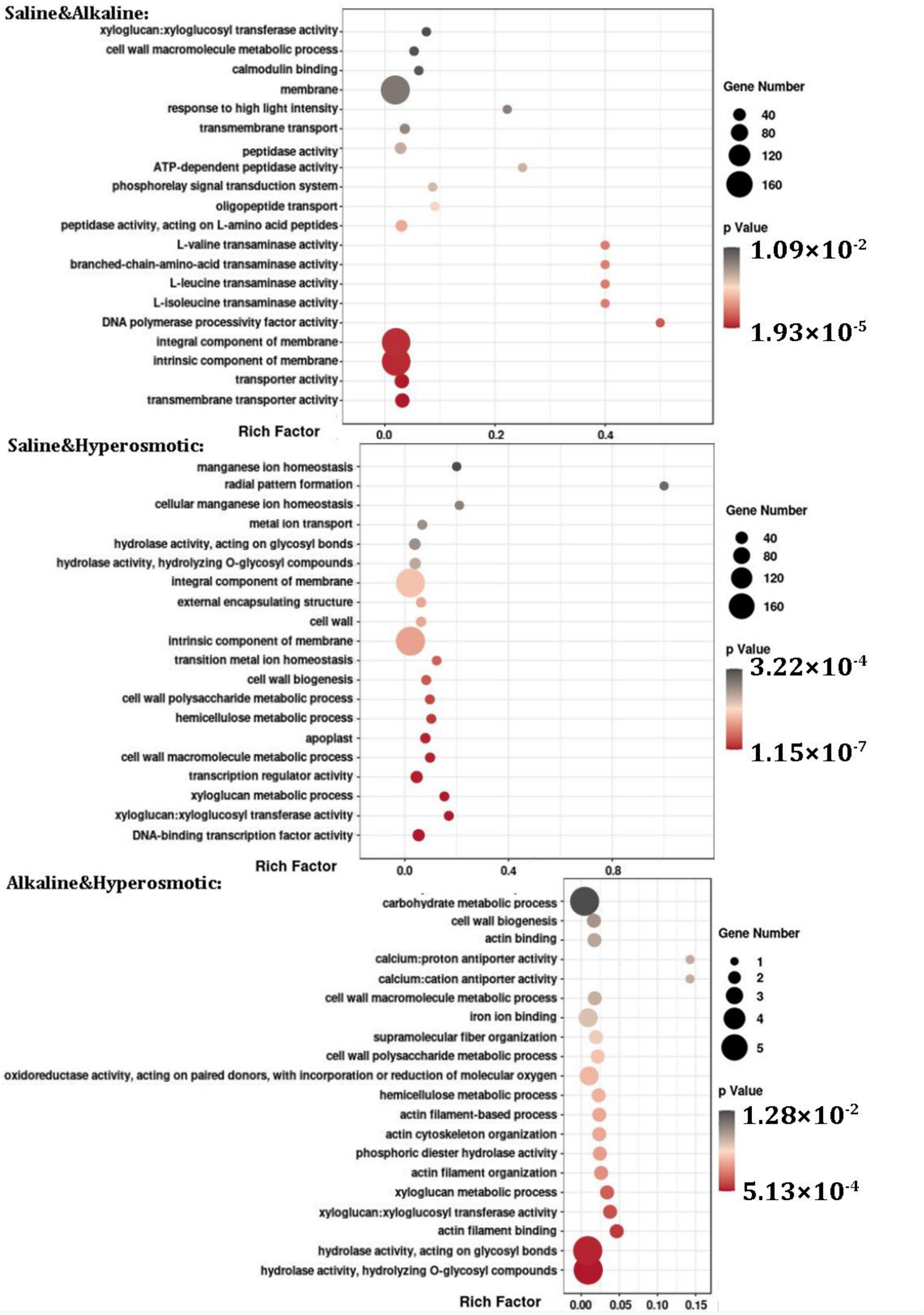

Figure S3. GO enrichment analysis of DEGs responded to double stresses.

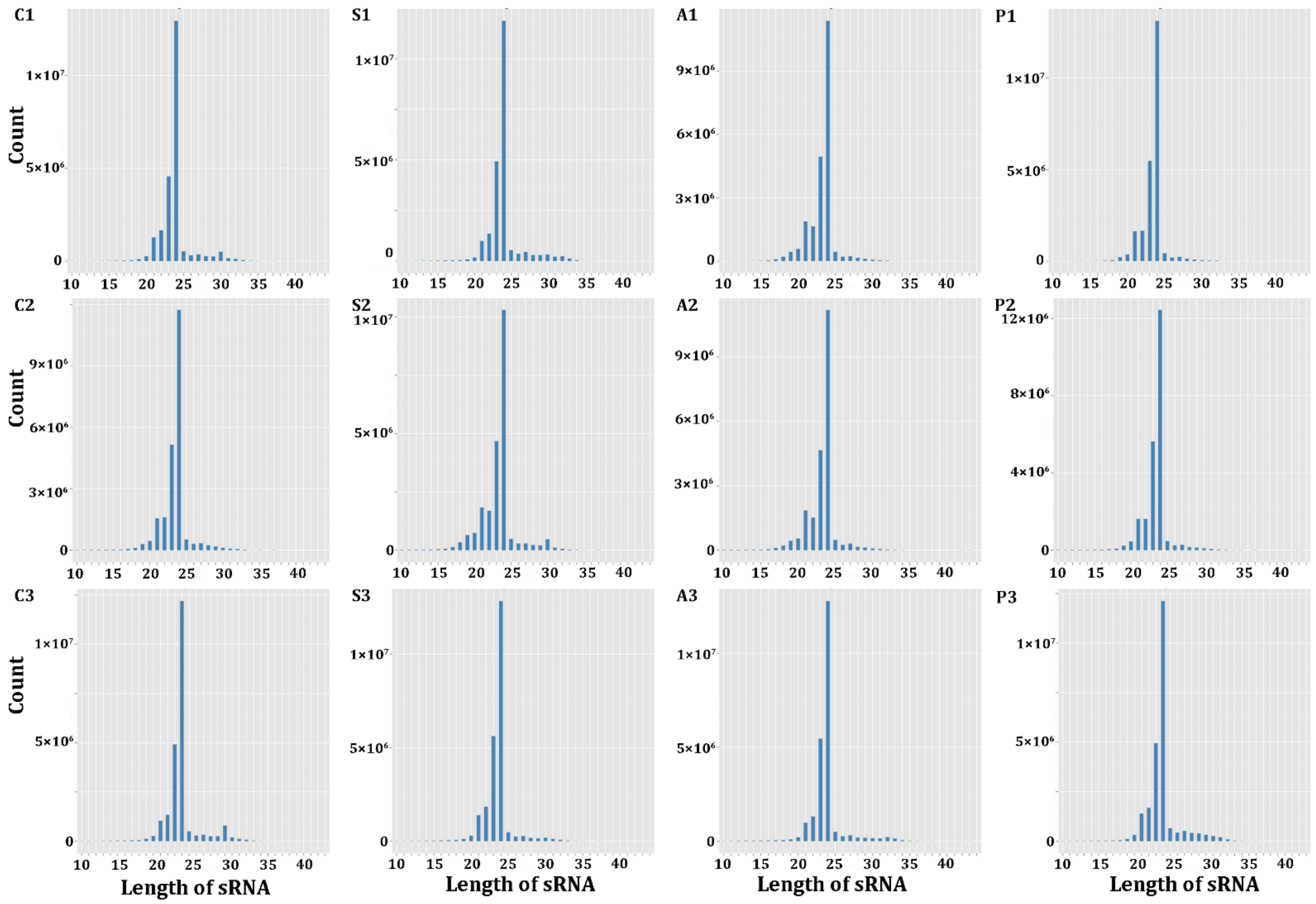

Figure S4. Length distribution of reads in small RNA sequencing.

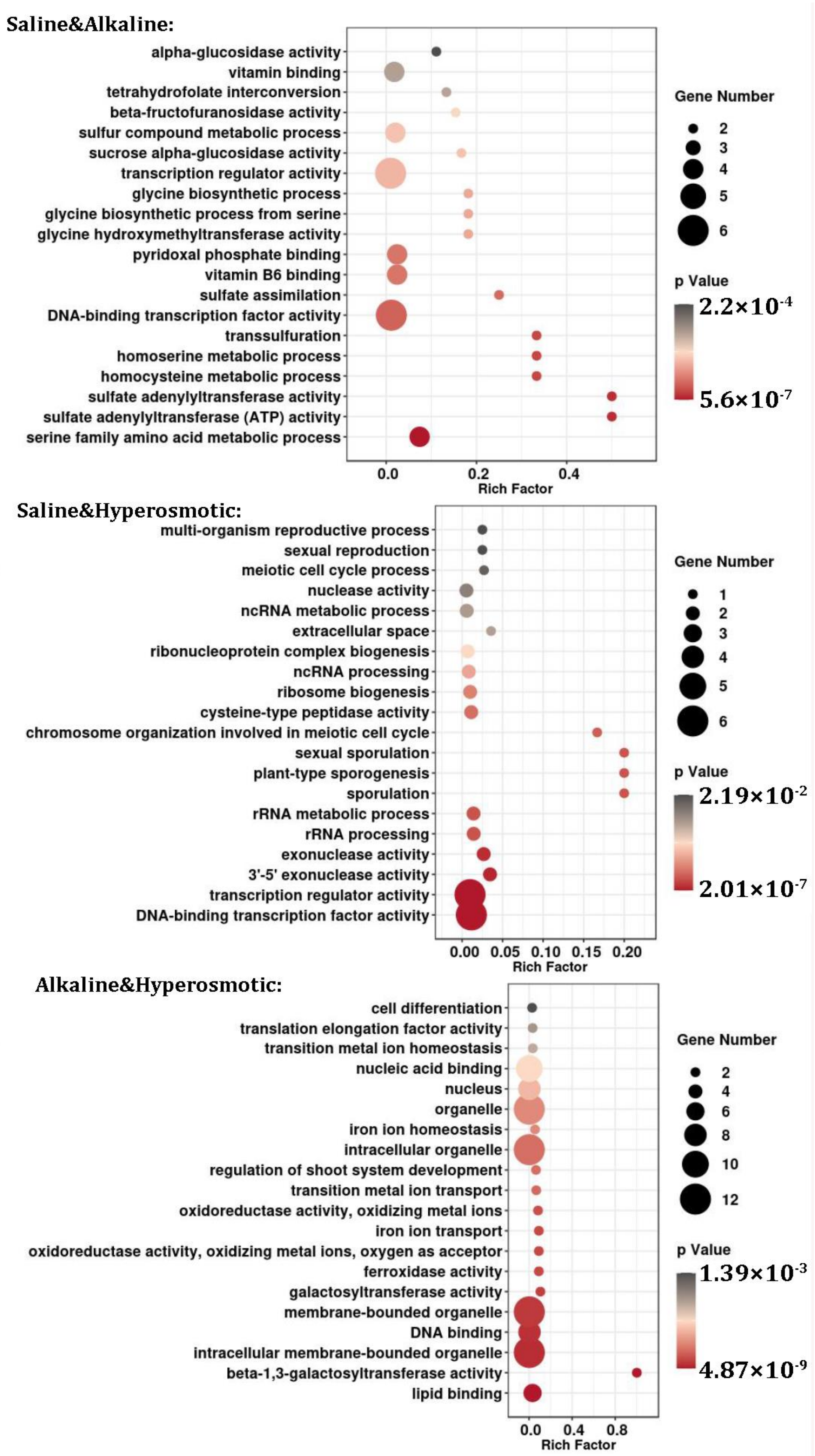

Figure S5. GO enrichment analysis of DEM targets responded to double stresses.
