# Supplementary figures and images for: Transcriptome and Small RNA Sequencing Reveals the Basis of Response to Salinity, Alkalinity and Hypertonia in Quinoa (Chenopodium quinoa Willd.)
Source: Int J Mol Sci. 2023 Jul 22;24(14):11789. doi: 10.3390/ijms241411789 (PMC10380837; doi:10.3390/ijms241411789)

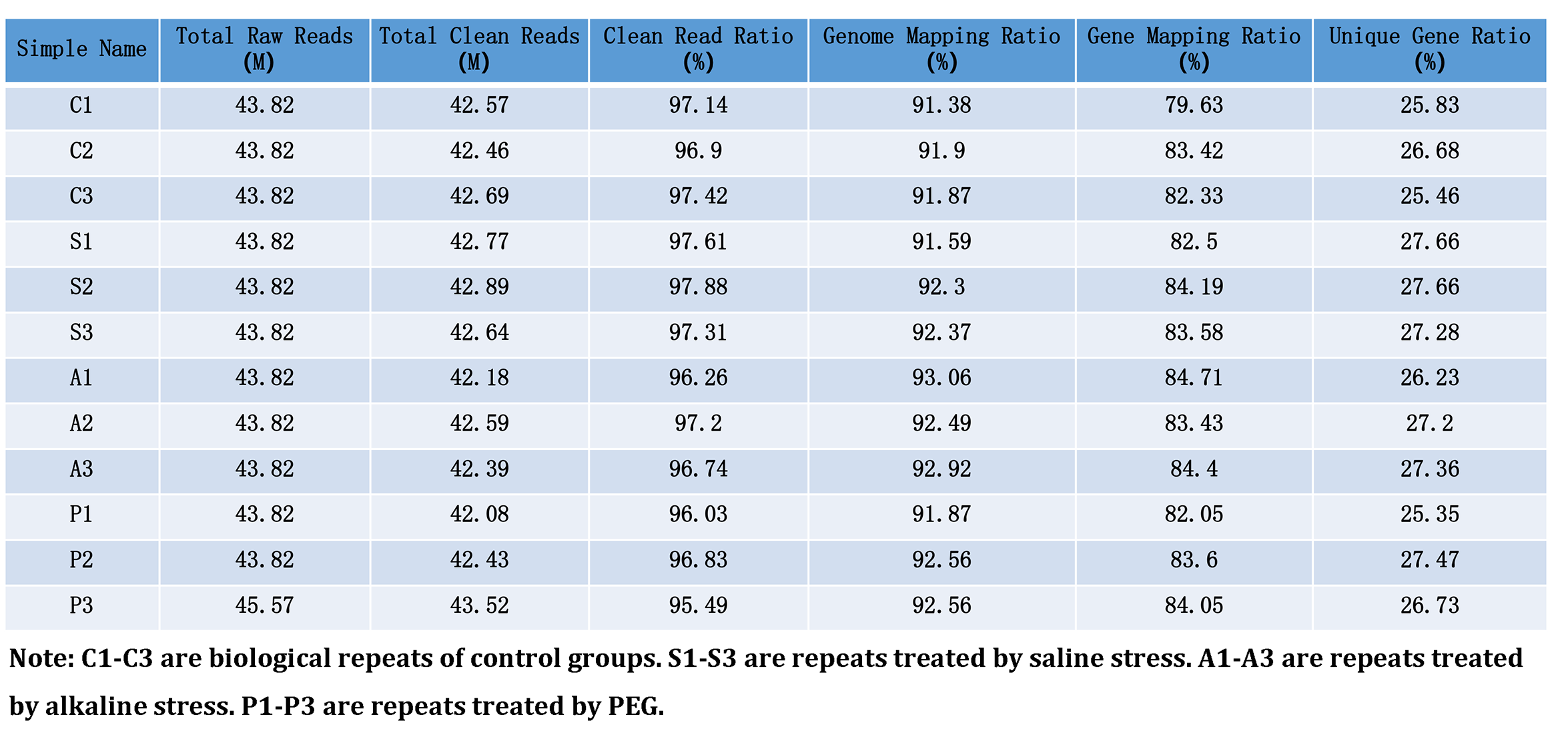

Supplement: Supplementary file 1 [file ijms-24-11789-s001.zip › Tab.S1 Statistical analysis of sequencing reads.tif]
